# Neo-Salvarsan for Scarlet Fever

**Published:** 1914-05-09

**Authors:** 


### Neo-salvarsan for Scarlet Fever.

In cases of scarlet fever a positive Wassermann reaction is not, of course, very uncommon. Hence the idea has occurred to several German physicians to try the effect of neo-salvarsan in the treatment of this disease. References to conclusions and to independent work are published by Dr. Fischer in the *Archives of Pediatrics* in a preliminary report upon his results. This observer began by trying neo-salvarsan only for those severe cases of septic scarlet fever in which a fatal issue is almost inevitable. The dose employed was 0.2 grams in 40 c.c. of plain sterile water, given intravenously. Five children received the treatment, varying in age from six months to nine years. All five were in an apparently hopeless condition when the neo-salvarsan was given. Three died, one recovered, and the fifth was still alive, but seriously ill, at the date of the report. Dr. Fischer is satisfied that no harm was done in any of the cases; no reaction, shock, or rash followed the injections. It is too early, he says, to be prejudiced for or against neo-salvarsan in septic scarlet fever, but it merits an extensive trial.

